# A Challenging Surgical Decision in a Child With Bowel Obstruction Secondary to Abdominal Tuberculosis and a Literature Review

**DOI:** 10.7759/cureus.33845

**Published:** 2023-01-16

**Authors:** Mohammad T Almohaidly, Tuqa A Alsinan, Lubabah Mohamadalmoktar, Nawraa A Alsinan

**Affiliations:** 1 Department of Pediatric Surgery, Prince Sultan Military Medical City, Riyadh, SAU; 2 Graduate Medical Education, Alfaisal University College of Medicine, Riyadh, SAU; 3 College of Medicine, Taibah University, Medina, SAU; 4 Medical Student, Alfaisal University College of Medicine, Riyadh, SAU

**Keywords:** exploratory lapratomy, pediatric surgery, enterocutaneous fistula, abdominal tuberculosis with intestinal obstruction, bowel obstruction

## Abstract

Tuberculosis (TB) is an infectious disease and one of the top 10 causes of death worldwide. Abdominal TB (ATB) can involve the peritoneum, lymph nodes, luminal structures, and solid organs, with a predominance of intestinal and peritoneal forms of the disease. Most pediatric cases may present with peritoneal and lymph node disease. This case reports a five-year-old girl who is medically and surgically free. She had ATB complicated with a bowel obstruction that resulted in an eventful outcome of fistula formation. The family gave a travel history to Egypt two months prior to the emergency first presentation. ATB is considered a severe and challenging infectious disease that affects several systems. It is associated with high mortality and morbidity rates, specifically in the pediatric population. This case discusses the importance of considering the possible complications of ATB in pediatrics to overcome unfavorable outcomes.

## Introduction

Tuberculosis (TB) is an infectious disease caused by Mycobacterium tuberculosis. It is a communicable disease that is one of the top 10 causes of death worldwide [1,2]. According to the Global Tuberculosis report 2022 by the World Health Organization (WHO), before the COVID-19 pandemic, TB was the leading cause of death from a single infectious agent [3]. Around 20% of all TB cases are extrapulmonary, with the abdominal form accounting for 10% [4]. Abdominal TB may arise alone or in association with pulmonary tuberculosis. It can involve the peritoneum, lymph nodes, luminal structures, and solid organs, with the predominance of intestinal and peritoneal forms of the disease [5]. Pediatric cases account for 10-20% of abdominal TB; most may present with peritoneal and lymph node disease, unlike adults, where intestinal TB predominates [6]. Mainly it can be managed successfully with anti-TB therapy [5]. Surgical intervention is usually preserved when complications arise from adhesions and inflammation, such as bowel perforation, obstruction, fistula formation, abscesses, and hemorrhage [7]. Here we report a case of pediatric abdominal TB complicated by bowel obstruction with an eventful outcome of fistula formation.

## Case presentation

A five-year-old girl with no medical or surgical history presented to the Emergency Department (ED) with a history of abdominal pain associated with distention and fever for one month. There was a significant unintentional weight loss of up to 3 kg. She had a positive travel history to Egypt two months ago, where the symptoms initially started, and she sought medical advice without improvement. Since then, she has had continuous abdominal pain with fever episodes. She was admitted under general pediatrics for further investigations and management. Upon physical examination, she did not look well, with a fever of 39ºC and vital signs were within normal limits. Her abdominal exam showed moderate distension with generalized tenderness, which was more remarkable on the right lower quadrant and associated with dullness on percussion.

As initial management, the patient was resuscitated with intravenous fluid, broad-spectrum antibiotics, and analgesia. Her laboratory work showed a low hemoglobin level (9 mg/dL), and no leukocytosis, with her average blood gas analysis. There was an elevation in C-reactive protein (75 ml/L), procalcitonin (0.25 mcg/l), and a positive QuantiFERON test. However, the rest of her laboratory workup was within normal limits, including liver and renal function tests. An abdominal ultrasound was unremarkable.

On the other hand, a Computed Tomography (CT) demonstrated diffuse peritoneal thickening, nodularity, and soft tissue deposits. Numerous enlarged mesenteric root lymph nodes with inguinal soft tissue thickening and free fluid on the right side. No bowel obstruction was detected. 

Interventional radiology was consulted for taking a peritoneal lymph node biopsy which showed a necrotizing granuloma and giant cells, indicating an ongoing inflammatory process. 

At that time, several differential diagnoses were considered and at the top of them were extrapulmonary tuberculosis, inflammatory bowel disease, infectious causes such as yeast and fungal, ovarian conditions, and primary metastatic malignancy. Unfortunately, the family signed a discharge against medical advice before the medical team finalized their diagnosis.

**Figure 1:**
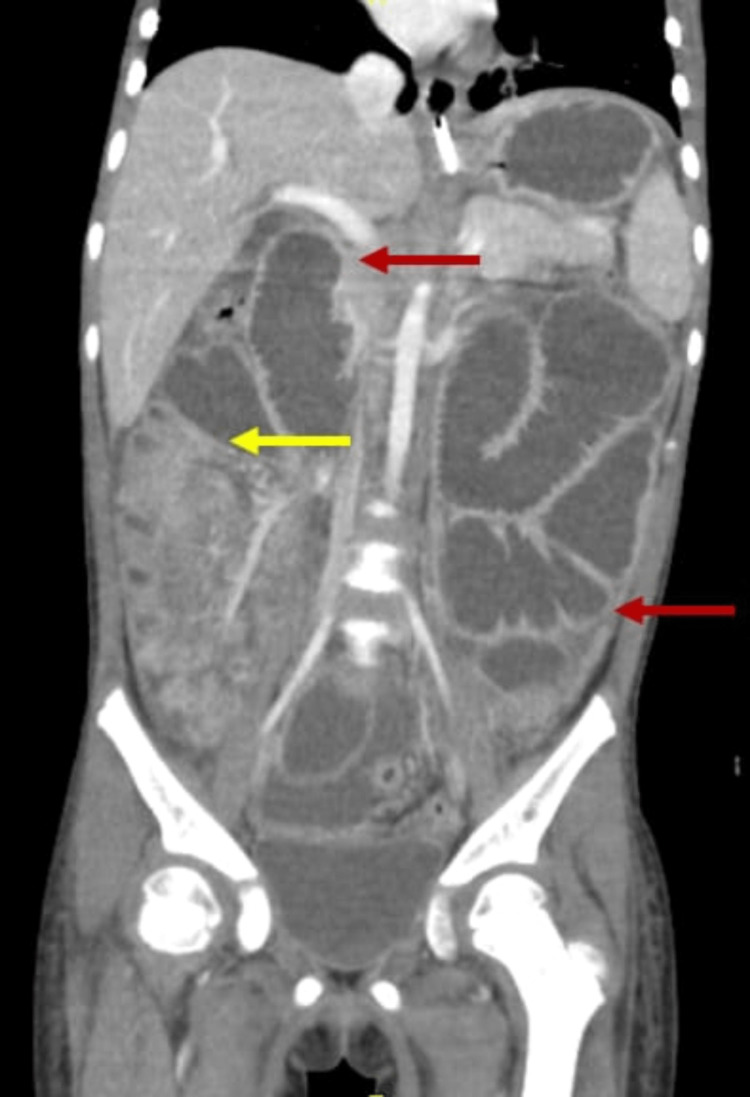
Coronal plane of repeated CT scan at second Emergency Department presentation showing a diffuse dilatation of proximal bowel (red arrows), and collapsed distal bowel. Distal bowel obstruction with transitional zone in the distal jejunum/proximal ileum (yellow arrow).

**Figure 2:**
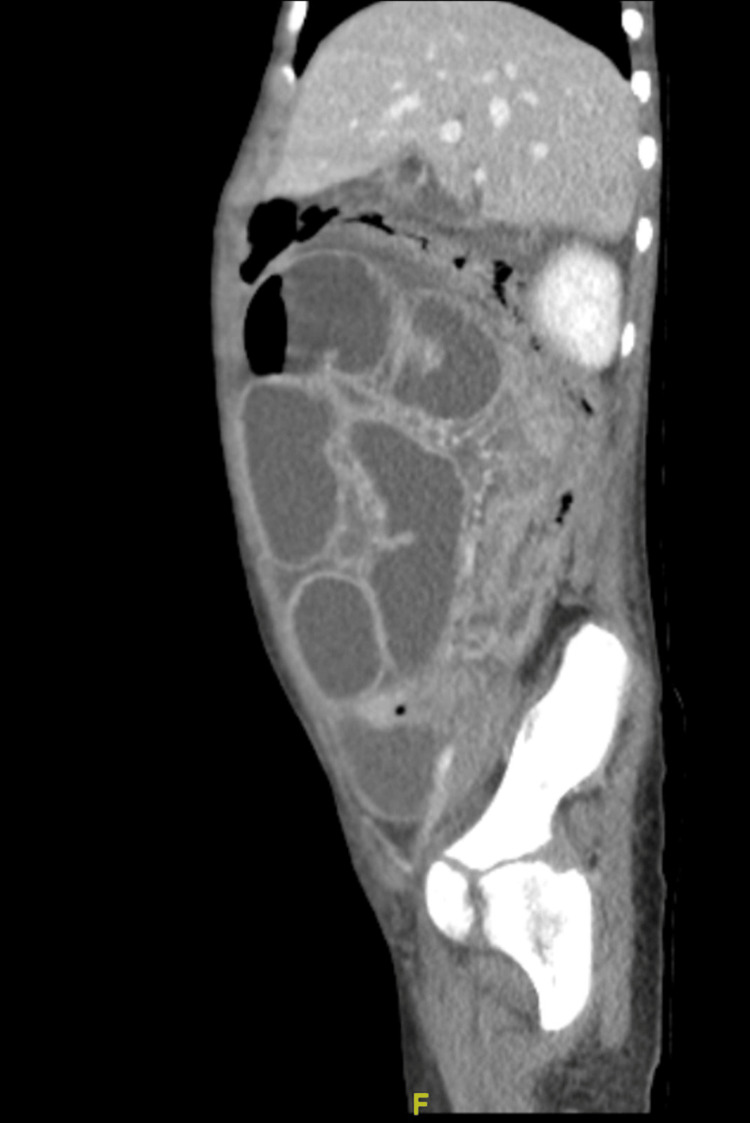
Sagittal plane of repeated CT scan of second Emergency Department presentation.

**Figure 3:**
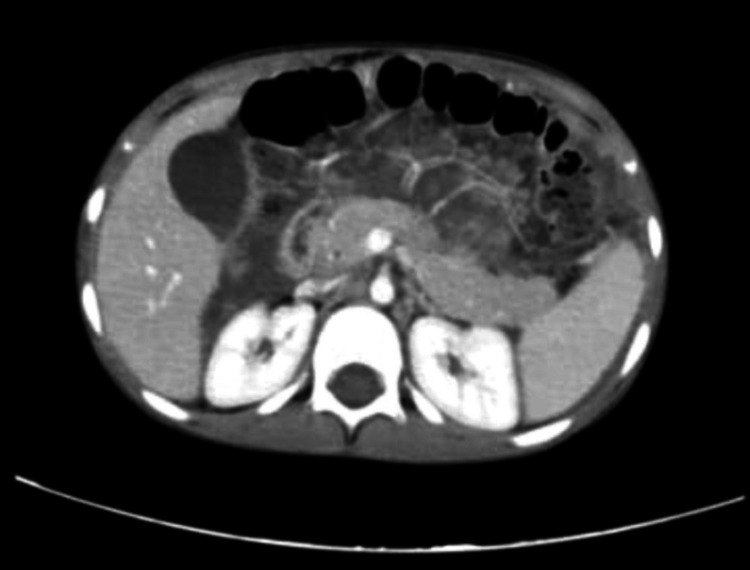
Axial plane of repeated CT scan of second Emergency Department presentation.

Two days after discharge, she presented to ED with a picture of bowel obstruction in terms of abdominal distention and pain, with projectile bilious vomiting. She looked ill, dehydrated, pale, tachycardic, and distressed upon examination. An abdominal exam revealed tension, tenderness, and a significant destination. CT was repeated, demonstrating a diffuse dilatation of the stomach, duodenum, and most proximal bowel. The distal bowel collapsed and was displaced to the right lower abdomen, including the whole colon. Distal bowel obstruction with a transition zone in the distal jejunum/proximal ileum was detected (Figures 1-3). 

**Figure 4:**
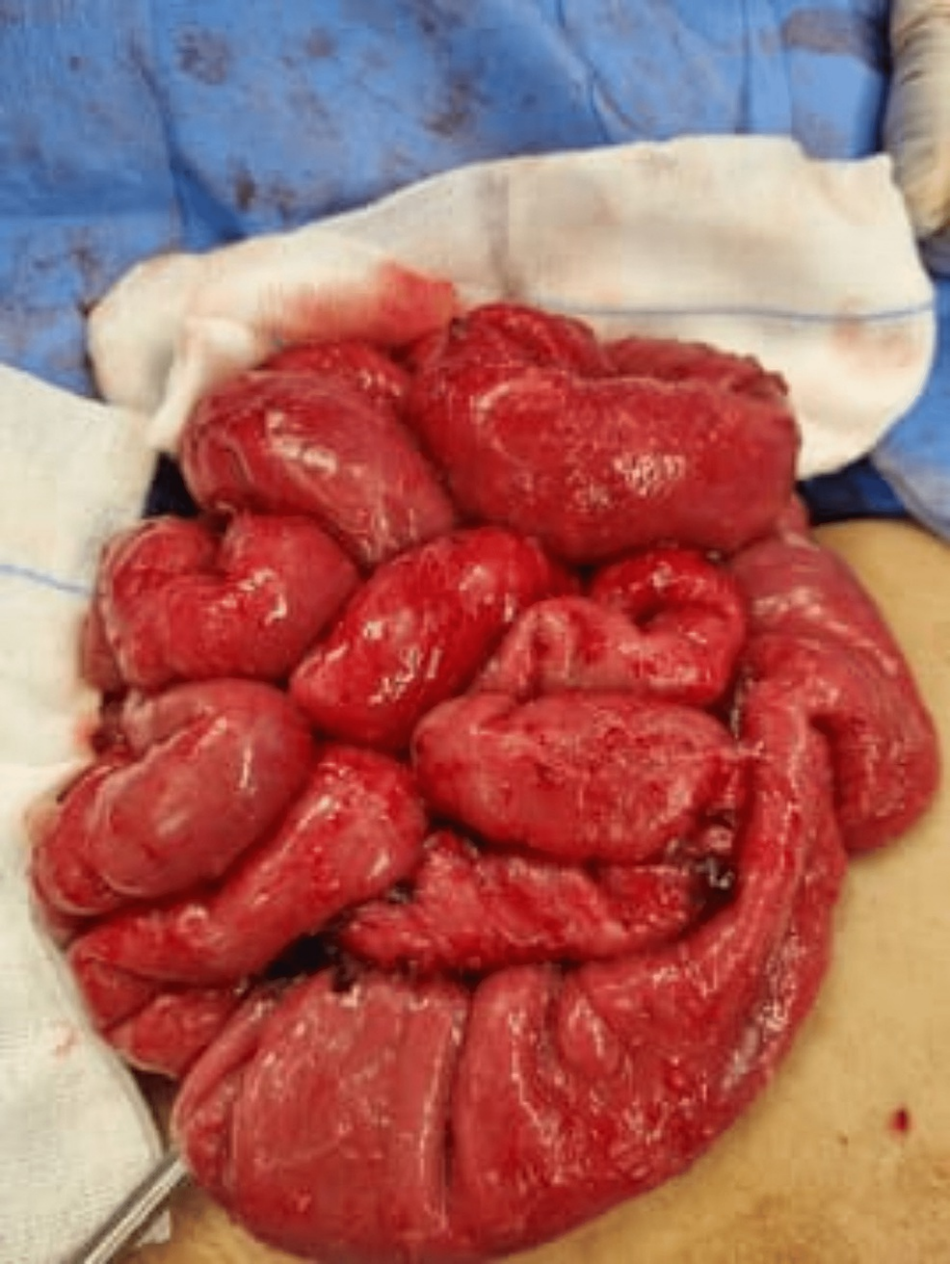
Intraoperative findings of severe adhesions, peritoneal thickening with extensive nodularity.

Pediatric surgeons were consulted in this presentation, and an exploratory laparotomy was arranged. Intraoperative findings showed significant ascites, severe adhesions, peritoneal thickening, extensive nodularity, and multiple enlarged lymph nodes with cheesy discharge. The transitional zone obstruction was found around the distal jejunum (Figure 4). 

**Figure 5:**
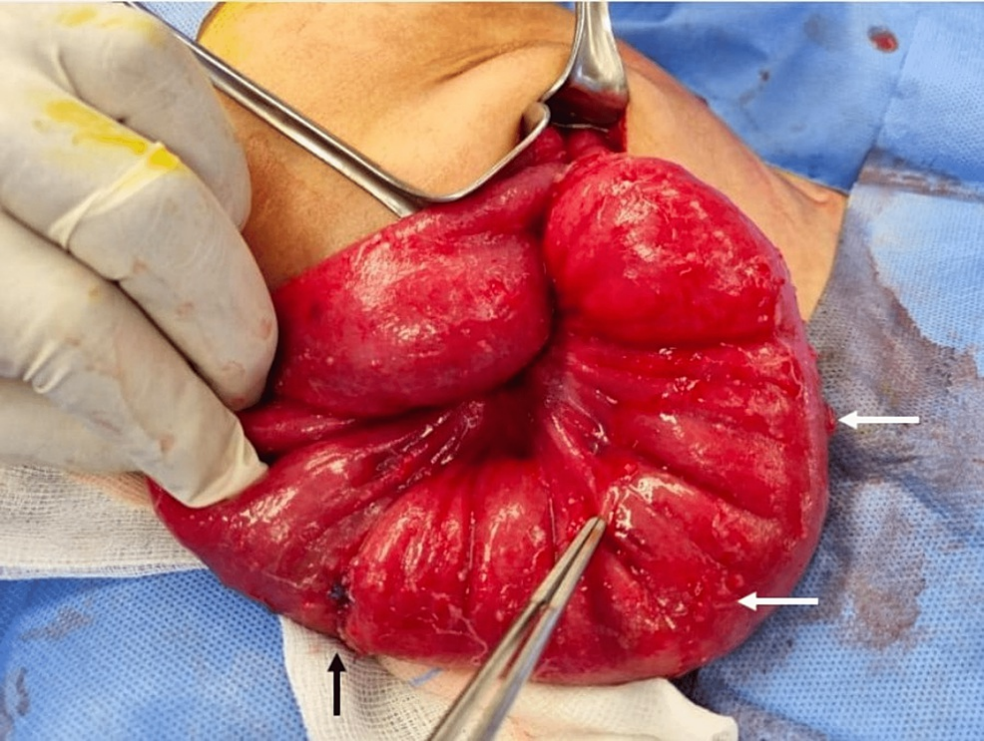
An iatrogenic bowel injury due to severe adhesions that was repaired primarily (black arrow). Extensive bowel nodularity was found (white arrows).

Mainly a release of adhesions was done with multiple biopsies taken from the peritoneum, bowel nodules, and lymph nodes which showed the same histological finding of necrotizing granulomatous inflammation. Unexpectedly, there was an iatrogenic bowel injury due to severe adhesions between the bowel and peritoneal wall that was repaired primarily (Figure 5). 

**Figure 6:**
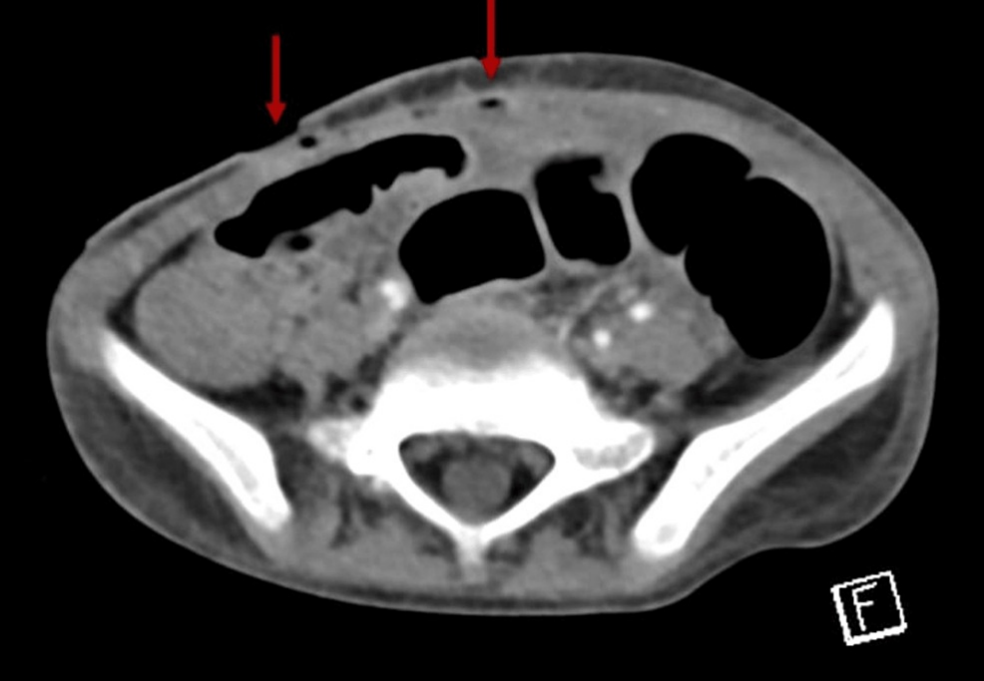
A Gastrografin study that reported an enterocutaneous fistula.

Postoperatively, the patient was admitted to the Intensive Care Unit (ICU) for 24 hours. She was kept on nil per os (NPO), started on total parenteral nutrition (TPN), and maintained on nasogastric decompression, broad-spectrum antibiotics, and anti-tuberculous therapy with continuous evaluation and reassessment by the pediatric surgery team. During the ninth day postoperative, the dressing was soaked and removed, which showed a purulent yellowish to greenish discharge from the lateral edge of the wound. We proceeded with a Gastrografin study that reported an enterocutaneous fistula finding confirming our impression with high output (300 ml/24 hour) (Figure 6).

The fistula was managed conservatively by keeping the patient NPO and on TPN. An octreotide medication was started, which resulted in a gradual decrease in fistula output, and it was closed after one month. The patient lost her follow-up visits to our clinics.

## Discussion

TB remains a challenging infectious disease, mainly if it affects organs other than the pulmonary system. Several studies were conducted in the literature discussing the management of extrapulmonary TB in the pediatric population. The diagnosis of abdominal TB remains challenging despite the multiple modalities available. The gold standard of diagnosis is still microbiological diagnosis with culture-positive results. Complete resolution of TB infection requires strict compliance with TB treatment [6]. 

Two consecutive studies were published in 2018 and 2020 in India and Pakistan, respectively. Endemic countries with TB are considered a risk factor for citizens and visitors. The reported cases who underwent a surgical intervention resulted in a fair outcome that was concluded it should be considered in patients with recurrent intestinal obstruction or pain, intestinal perforation, or gastrointestinal bleeding. Moreover, determining the abdominal TB presentation, its surgical procedures, and outcomes in children showed that perforation and obstruction could occur during or after the completion of abdominal tuberculosis treatment (ATT). As a result, management requires early recognition with possible medical treatment and surgical intervention, as indicated [1,4]. 

In addition, understanding the detailed clinicopathological profile and outcomes of surgical management of abdominal tuberculosis (ATB) would help overcome any diagnostic delay, such as in patients who present late with intestinal obstruction. ATB is a disease with high mortality and mimics inflammatory bowel disease and small bowel tumors, which requires a high index of clinical suspicion by reducing the morbidity and mortality from this disease, especially in parts of the world where TB is endemic [2,5]. The extrapulmonary manifestation of ATB in pediatric patients often presents with fever, abdominal pain, and distention, with ileocecal tuberculosis the most common site of involvement in bowel obstruction [5,8].

Several diagnostic approaches could be followed for ATB symptomatic patients as a combination of investigations that could be helpful to both aid diagnosis and define the extent of the disease. Abdominal ultrasound should be used more frequently in children with possible TB and any abdominal symptoms [7]. Furthermore, a diagnostic algorithm was discussed in a recent systematic review to help reach the proper diagnosis by histopathology or microbiology. It was concluded that it needs unique expertise, which is rarely used in low- and middle-income countries. Also, capsule endoscopy may cause complete intestinal obstruction in small bowel strictures. Only 80% of the patients can reach the final diagnosis and start therapeutic management [8]. Another comprehensive review of imaging manifestations of ATB and its mimics was conducted in the year 2021 in Qatar. This review shows that ATB is a significant public health concern worldwide, affecting virtually every organ system. A high index of clinical suspicion and knowledge about the appearance of TB infection within various organ systems and its mimics is central to guiding appropriate, timely treatment, especially in high-risk populations such as pediatric patients [4].

Table 1 summarizes the literature about ATB in pediatrics describing the challenging aspect of this disease over the past years. 

**Table 1:** Literature review over the past years about challenging ATB cases and their outcomes. YRS; years. M; months. TB; tuberculosis. ATB; abdominal tuberculosis. ATT; antituberculosis therapy. ESR; erythrocyte sedimentation rate. CRP; C-reactive protein.

Year	Authors	Study Design	Age of Patient	Objective	Findings	Results
2022 [9]	Lukosiute-Urboniene et al.	Case series	5, 11, 15, and 16 yrs.	Reporting four cases of children with ATB.	The diagnosis was confirmed only after diagnostic laparoscopy and biopsy.	QuantiFERON test and ascitic fluid analysis should be performed before invasive interventions.
2021 [4]	Arbab et al.	Retrospective study	8-14 yrs.	Determining the presentation, surgical intervention, and outcomes.	14 cases underwent laparotomy. 4 children died of sepsis postoperatively.	Perforation and obstruction can occur during or after the completion of ATT.
2020 [10]	Wong et al.	Case series	8-14 yrs.	Examining the clinical profile and treatment outcomes of ATB.	2 out of 6 patients required emergency laparotomy.	ATB should be considered in children with chronic abdominal symptoms for at least 8 weeks with anemia, raised ESR, and CRP.
2020 [11]	Lal et al.	Retrospective study	0.25–12 yrs.	Presentation, the pattern of distribution, and diagnosis of ATB.	9 patients underwent surgery. 6 for perforation- Peritonitis. 3 for intestinal obstruction. 2 for endoscopic dilatation of intestinal strictures.	Microbiological confirmation is possible in only one-third of the patients.
2019 [12]	Kuo et al.	Case report	10 yrs.	Reporting case of ATB.	Underwent diagnostic laparoscopy.	Acid-fast smear and culture of ascitic fluid were not diagnostic.
2018 [13]	Zaslavsky et al.	Case report	9 and 3 yrs.	Presenting 2 pediatric cases of peritoneal TB with an insidious onset of symptoms.	1 patient underwent exploratory laparotomy.	CT findings are the key to establishing the diagnosis.
2017 [14]	Rodrigo et al.	Case report	6 yrs.	Presenting a case of multisystemic TB with intestinal involvement.	Exploratory laparotomy revealed multiple perforations secondary to obstructions in the ileum.	The patient received ATT for 1 y with a favorable outcome.
2017 [15]	Usta et al.	Retrospective study	7-16 yrs	Presenting 4 years experience on the clinical features of ATB in children.	Laparotomy was performed on 5 patients.	Laparoscopy /laparotomy could be useful, invasive methods with clinical suspicion may prevent delay of diagnosis.
2010 [16]	Tinsa et al.	Retrospective study	7-14 yrs.	Reviewing clinical features of ATB in children.	Diagnostic laparotomy was performed on 3 patients.	Laparoscopy in Histopathological diagnosis brought down the rate of unnecessary laparotomies.
2010 [17]	Lin et al.	Retrospective study	Mean (14.7 yrs), with 1 patient < 10 yrs old.	Describing the clinical and radiologic manifestations.	1 died due to disseminated disease. 3 underwent laparotomy. 1 had a laparoscopy.	Surgery is reserved for tissue diagnosis and managing complications.

## Conclusions

ATB is considered a severe and challenging infectious disease that affects several systems. It is associated with high mortality and morbidity rates, specifically in the pediatric population. This case reports a female child who had ATB complicated with a bowel obstruction that resulted in an eventful outcome of fistula formation. The positive travel history indicates a risk factor that could be managed by taking prophylaxis or considering it a differential diagnosis upon the first presentation. This case discusses the importance of considering ATB's possible complications in pediatrics to overcome unfavorable outcomes. 
